# Neuropathic Pain: Challenges and Opportunities

**DOI:** 10.3389/fpain.2020.00001

**Published:** 2020-08-07

**Authors:** Monique van Velzen, Albert Dahan, Marieke Niesters

**Affiliations:** Department of Anesthesiology, Leiden University Medical Center, Leiden, Netherlands

**Keywords:** neuropathic pain, pain, phenotype, central sensitization, pharmacotherapy

## The Burden of Neuropathic Pain

Chronic pain has a high prevalence in the Western World, where one in five individuals suffers from chronic pain [1], that we define as frequent painful episodes lasting for at least 3 months. A substantial number of these patients experience symptoms of neuropathic pain (30–40%) [2]. The International Association for the Study of Pain (IASP) defines neuropathic pain as “pain caused by a lesion or disease of the somatosensory nervous system” and can relate to the peripheral or central nervous system [3]. The presence of chronic pain has far-reaching consequences for patient, family members and society. Pain in general and neuropathic pain in particular negatively impact the patients' quality of life and ability to participate in common daily and work activities. Apart from personal suffering, chronic pain cost to society is significant and lies in the range of billions of dollars per year [4].

The origin of neuropathic pain is diverse and related to a large variety of often difficult to treat underlying diseases or lesions. For example, neuropathic pain may occur due to trauma to the central or peripheral nervous system (e.g., surgical trauma, spinal cord injury, complex regional pain syndrome), nerve compression, vascular disease (e.g., stroke), neurological diseases (e.g., multiple sclerosis, syringomyelia), infectious diseases (HIV, leprosy, shingles), metabolic syndromes (diabetic mellitus, sarcoidosis, alcoholism), drugs (e.g., chemotherapeutics) or hereditary syndromes (e.g., Fabry's disease, erythromelalgia, channelopathy). Additionally, neuropathic pain is common in cancer patients, in which the perceived pain often has mixed neuropathic and nociceptive components. Finally, in some patients the cause of the neuropathic pain symptoms is unknown. Given the above, the presence of the high variety in underlying processes responsible for neuropathic pain, with additionally all the patient variations expressed within single diseases, precede the notion that treatment will be difficult and should be individualized per patient.

The symptoms of neuropathic pain include localized or more widespread spontaneous pain, perceived as burning, electrical or shooting, often in combination with paresthesias. Physical examination may show skin areas that display allodynia (reduced pain threshold), hypoalgesia (increased pain threshold) or hyperalgesia (increased pain sensitivity) [5]. Allodynia and hyperalgesia are indications of central sensitization or a hyperactive response to normal or subthreshold afferent stimuli. Occurrence of central sensitization is a sign of an increase in disease severity. Measurement of the endogenous pain modulatory system and sensory testing, in which a variety of stimuli and tests are applied to the skin, indicate that several phenotypes of neuropathic pain exist independent of the underlying disease [5–7].

On top of symptoms of pain, the majority of neuropathic pain patients experience other symptoms such as alterations in mood (sometimes leading to depression), anxiety, sleep disorder, neurocognitive impairment (e.g., memory defects), generalized malaise and other often-ill described complaints [8]. It is our experience that most physicians do not consider these complaints serious enough or part of the pain syndrome to evaluate, diagnose or treat them. That is a missed opportunity since animal models of neuropathic pain point toward the existence of chronic pain-induced well-defined pathophysiological substrates in the brain of anxiety and memory impairment that may be an important target for intervention [9].

## Lack of Efficient Pharmacotherapy

In addition to the challenges related to the large number of patients that experience neuropathic pain symptoms, by far the most important challenge to doctors and scientists is the lack of adequate efficacy of currently available pharmacotherapy. Poor outcomes of randomized trials are translated into clinical practice where doctors and patients are painfully aware of the small effects of currently available treatments. Moreover, dose escalation of available treatments is often not possible due to the development of side effects that limit compliance to therapy. As discussed by Finnerup and colleagues [10], the absence of positive trials is most probably related to limited drug efficacy, large placebo responses, heterogenous diagnostic criteria, and poor patient phenotyping. Most relevant treatments available for neuropathic pain include antidepressants (tricyclic antidepressants, TCAs, selective noradrenaline reuptake inhibitors, SNRIs), gabapentinoids (pregabalin, gabapentin), sodium channel blockers (lidocaine, lacosamide), the TRPV1 agonist capsaicin and opioids. Less mainstream treatments include ketamine, cannabinoids and botulinum toxin [5, 11]. The number needed to treat (pain reduction by at least 50%, NNT) varies from 3.6 for the TCAs to 6.4 for the SNRIs, with numbers needed to harm (NNH) of 13.4 and 11.8, respectively (most important side effects are dizziness, orthostatic hypotension and sedation) [10]. One needs to realize that studies on TCAs are relatively old and due to the increasing placebo responses over time [12], the NNT for SNRIs increased substantially. Additionally, due to co-occurrence of depression and anxiety, at least part of the beneficial effects comes from mood improvement and reduced anxiety. Equivalent NNT and NNH numbers for pregabalin are NNT 7.7 (NNH 13.9), gabapentin NNT 7.2 (NNH 29), strong opioids NNT 4.3 (NNH 4.7), and weak opioids NNT 11.7 (NNH 12.6) [10]. In light of the quality of evidence, Finnerup et al. [10] give strong recommendations for the TCAs and gabapentinoids and weak recommendations for opioids and patches with lidocaine or capsaicin. Still, recent studies by Martini et al. [13, 14] in patients with postherpetic neuralgia or diabetic polyneuropathy, treated with a capsaicin 8% patch vs. an active control (capsaicin 0.04%), show four to five distinct responder groups depending on analgesic efficacy including a group of non-responders and a group showing a full analgesic response. Relative to the active placebo group, there were 40% less patients in the non-responder group and 25% more in the full response group (*p* < 0.01). These results indicate that despite moderate treatment efficacy in the full population of neuropathic pain patients, high efficacy is possible in well-defined subsets of patients, while others do not respond, notwithstanding a similar presumed underlying etiology of their neuropathic pain symptoms. It is challenging to *a priori* identify those patients that might respond to specific treatment. Evidently, these subgroups differ on many levels, such as differences in disease characteristics and severity (with differences in duration of disease, central neuroinflammation, central sensitization, activity of the endogenous pain system, baseline pain sensitivity), comorbidities, comedication use (causing pharmacokinetic and pharmacodynamic interactions with analgesic medication), genetic background, psychological make-up, social status, etc. Interestingly, the study by Martini et al. [13] showed that prior treatment with an opioid had a negative predictive value on efficacy of the high-dose capsaicin patch.

## Opportunities

An important question is whether we can transform the challenges that we encounter in daily practice into opportunities, aimed at improving treatment efficacy and consequently reducing patient suffering. For example, major advances are possible when improved and more sensitive diagnostic tools become available, new medication or repurposed existing drugs that show high analgesic efficacy combined with reduced adverse effects are being admitted to the market, and, most importantly, if we are able to provide precise and personal pain therapies based on patient characteristics as outlined above rather than based on the underlying disease or the presence of neuropathic pain symptoms *per se*.

### Cornea Confocal Microscopy

A recent new development in the application of novel diagnostic tools is the use of confocal cornea microscopy (CCM) in the non-invasive diagnosis of small fiber neuropathy [15]. CCM is considered a surrogate marker of small fiber pathology in peripheral neuropathies and has been used to detect small fiber damage in patients with diabetes mellitus, sarcoidosis, fibromyalgia, and Fabry's disease, to name a few [15–18]. The strength of the technique is its simplicity, ability to reuse repetitively over time without causing tissue damage and rapid availability of results. Additionally, it can be used to monitor the modulatory effects of treatment. For example, in patients with type 1 diabetes mellitus and end stage renal failure, cornea C-fiber density and cornea C-fiber length increased after kidney and pancreas transplantation with concomitant improvement of neuropathy symptoms, compared to a group of patients with type 1 diabetes mellitus and severe polyneuropathy without transplantation surgery [19]. Similar results were obtained in patients with sarcoidosis-associated small fiber loss and neuropathic pain following treatment with cibinetide (ARA290), a small peptide acting at the innate repair receptor [20]. These data indicate that the non-invasive technique of CCM can be used to diagnose small fiber abnormalities in neuropathic pain patients and detect early regeneration of C-fibers in the cornea.

### Pharmacotherapy

A large number of new pharmacological treatments are currently under consideration and are based on molecular targets with evidence of involvement in neuropathic pain [21]. These targets include for example opioid receptors, voltage-gated sodium channels, calcium channels, *N*-methyl-D-aspartate (NMDA) receptors, monoamine transporters, cannabinoid receptors, TRPV1 receptors, and the innate repair receptor [22]. It is important that new drugs not only produce effective pain relief but concurrently have a superior side effect profile compared to existing drugs of the same class. One example is the experimental drug cebranopadol, an opioid acting at the mu-opioid receptor and the nociceptin/orphanin FQ peptide (NOP) receptor. Cebranopadol was effective in animal models of neuropathic pain [23]. To assess the safety of cebranopadol, we developed safety or utility functions. These functions allow the simultaneous assessment of benefit and harm of an analgesic by creation of a single function that considers probability of analgesia and (in this case) respiratory depression as function of time (after single or multiple administrations) or as function of the concentration at the effect site [24]. Our results indicate an advantage of cebranopadol over classical mu-opioid agonists considering analgesia and respiratory toxicity, most probably related to its activity at the NOP receptor. This is highly relevant given the current opioid epidemic and surge in opioid fatalities [25]. The utility function is a general concept that can be applied to determine the safety of any analgesic considering multiple endpoints [26]. For example, in patients with chronic pancreatitis treated with pregabalin, the utility function showed an increase of the probability of analgesia over time relative to the probability of severe dizziness [27], suggestive that it is worthwhile to maintain pregabalin therapy in this complex patient group despite early onset toxicity.

Other treatments that we find of interest are the psychedelics cannabis and ketamine. The endocannabinoid system is involved in neuropathic pain control and cannabinoid receptors are expressed on neurons and immune cells [28]. In an exploratory placebo-controlled study, we recently showed that inhaled Δ^9^-tetrahydrocannabinol but not cannabidiol (CBD) may be an effective treatment in fibromyalgia patients, with little toxicity apart from mild drug high sensations [29]. Although about 50% of these patients had symptoms of small fiber damage, future studies should address the efficacy and toxicity (including abuse potential) of THC and CBD in patients with established neuropathy. The NMDA receptor plays an important role in the chronification of pain and maintenance of neuropathic pain symptoms. Low-dose (subanesthetic) ketamine is more and more successfully used to manage treatment-refractory neuropathic pain by physicians in second and third line. The choice for ketamine seems reasonable given the fact that it decreases windup and temporal summation, surrogate measures of central sensitization [30–32]. Still, most if not all randomized controlled trials on the efficacy of ketamine in the treatment of neuropathic pain yielded negative results [33]. This indicates the lack of proof of benefit (rather than the lack of benefit) [34], and additionally is a sign that classical randomized trials may not be able to detect the potential benefit of ketamine in neuropathic pain syndromes. As argued earlier, ketamine may well be effective in patients with a specific neuropathic pain phenotype (e.g., in patients that display symptoms of hyperalgesia and/or allodynia, patients with a defective endogenous pain modulatory system, patients with a concomitant mood disorder) rather than in the general, more heterogeneous neuropathic pain population [35].

### Patient Phenotyping

Various authors agree that phenotyping or stratifying of patients into well-described subgroups by sensory profiling and by use of other tools and biomarkers (e.g., by CCM, questionnaires, patient history; see above) will allow us to improve our knowledge of the disease process, get an appreciation of the severity and varieties of the disease process and will allow personalized choice of treatment [5, 6, 17]. While at present patient management is characterized by a trial-and-error approach, this new practice will shorten the time to effective treatment. One recent example of the application of phenotyping in drug treatment is our observation of the decrease in segmental sensitization and pain in patients with chronic low back pain and reduced or absent conditioned pain modulation (a surrogate marker of the endogenous pain modulatory system) during treatment with the bifunctional opioid tapentadol (a μ-opioid receptor agonist with additional inhibition of neuronal noradrenaline reuptake) (unpublished observation). This is evidence that the endogenous pain modulatory system may be applied as biomarker for individualized, personalized and mechanism-based treatments of low back pain patients with signs of sensitization. Existence of multiple pain phenotypes is now established in fibromyalgia, low back pain and peripheral neuropathy [6, 17]. The challenge remains to determine which treatments are the best choice for the various phenotypes.

In summary, neuropathic pain is present in ~1 in every 20 citizens in the Western World and is a major cause of suffering and burden to society. We expect that these negative consequences will increase in the world due to the COVID-19 pandemic related shrinking of financial resources available to the chronic pain population in general and the neuropathic pain population in particular. We here give a picture of challenges and opportunities that may serve as a guideline toward improved care of the neuropathic pain patient. Especially the concept of patient phenotyping is attractive as it will allow a more precise and personalized approach toward the patients with a complex neuropathic pain syndrome. Phenotyping will enable us to disentangle the various components of disease independent of the underlying etiology and will direct us toward treatment of each of the components. Examples are treatment of defects in endogenous pain modulation or central (or segmental) sensitization by existing or novel drugs that are specifically aimed at these components.

## Author Contributions

All authors participated in the inception, writing, and commenting of this editorial. All authors approved the paper.

## Conflict of Interest

The authors declare that the research was conducted in the absence of any commercial or financial relationships that could be construed as a potential conflict of interest.
